# Review of *Infectious Disease Ecology: Effects of Ecosystems on Disease and of Disease on Ecosystems* by Richard S. Ostfeld, Felicia Keesing, and Valerie T. Eviner

**DOI:** 10.1186/1756-3305-1-28

**Published:** 2008-08-29

**Authors:** Jean-François Guégan

**Affiliations:** 1 Genetics and Evolution of Infectious Diseases, IRD, Montpellier, France; 2 French Center on Globalization and Infectious Diseases, French School of Public Health, Montpellier, France

## Review

*We all as humans stay at the parasites' place which in turn reside in us! Nous demeurons chez des microbes qui nous habitent *!

Are we totally wrong in thinking that we are, as humans, like all other living organisms on Earth, (just) hosts and resources for parasites! On the contrary, would we be just staying at a parasites' home, since that our point of view on parasites and pathogens is totally flawed by an anthropogenic, too medically-oriented perspective? At a time where we are demonstrating that the human genome is not a single-formed entity but it is made for a substantial part of past genetic interactions between environmentally-resident pathogens and our species, the book co-edited by Richard S. Ostfeld, Felicia Keesing, and Valerie T. Eviner comes at just the right moment in offering some refreshing ideas and opening new research avenues on the exact role played by parasites in ecosystems, and *vice versa*. The book is composed of four parts – Part I, *Effects of Ecosystems on Disease*; Part II, *Effects of Disease on Ecosystems*; Part III, *Management and Applications*; Part IV, *Concluding Comments: Frontiers in the Ecology of Infectious Diseases*. It puts together 22 different chapters, most having being written by the more talented and influential research individuals in the fields of ecology of infectious diseases and disease management. People more interested in vectors and parasites will find some important reconsiderations on vector-parasite interactions since, in reality, many parasites may be capable of adapting on multiple vector species *en route *for transmission and many vectors may support multiple pathogens as well. With this book, following a series of some previous contributions, we are definitely entering into a new research dimension in which parasites are not just considered as killers that might be fought, not put at the centre of all Earth ecosystems organization, but put at their right place: they constitute an important component of biological diversity and organization. Some new ideas and theories exposed in the book, like the development of adequate models to deal with complex systems, the extension of the Red Queen hypothesis at a community-scale level or of the resilience theory to socioecological systems, and the application of ecological theories to disease management and policies, will be of particular interest to the reader. I did appreciate reading this important masterpiece of ecology of infectious diseases' science, which in its Part III opens a necessary and important discussion, that still needs to be further pursued, with management- and policy-makers (I am not totally convinced that it is to only research ecologists to make the first step in approaching these categories of professionals, but it is a two-way process as ecology informs us!). Time is gone to reconsider our viewpoint on parasites and to help every citizen understanding the basic linkages between ecosystems, diseases and health. Again, micro-organisms and others viruses and prions are not just like enemies in ecosystems, but they constitute for sure the bulk of living diversity on the planet, thus contributing to its organization and evolution.

## Competing interests

The author declares that they have no competing interests.

